# In Vitro and In Ovo Evaluation of *Oenothera biennis* L. Oil as an Alternative Preservative for Oil-Based Products

**DOI:** 10.3390/foods14020332

**Published:** 2025-01-20

**Authors:** Ramona Fecker, Ștefana Avram, Ileana Cocan, Ersilia Alexa, Larisa Bora, Daliana Minda, Ioana Zinuca Magyari-Pavel, Cristina Adriana Dehelean, Corina Danciu

**Affiliations:** 1 Department of Pharmacognosy, Faculty of Pharmacy, “Victor Babes” University of Medicine and Pharmacy Timişoara, Eftimie Murgu Square, No. 2, 300041 Timişoara, Romania; ramona.fecker@umft.ro (R.F.); larisa.bora@umft.ro (L.B.); daliana.minda@umft.ro (D.M.); ioanaz.pavel@umft.ro (I.Z.M.-P.); corina.danciu@umft.ro (C.D.); 2 Research and Processing Center of Medicinal and Aromatic Plants, “Victor Babes” University of Medicine and Pharmacy, Eftimie Murgu Square, No. 2, 300041 Timişoara, Romania; 3 Faculty of Food Engineering, University of Life Sciences “King Michael I” Timişoara, Calea Aradului 119, 300645 Timişoara, Romania; ileanacocan@usvt.ro (I.C.); ersiliaalexa@usvt.ro (E.A.); 4 Department of Toxicology, Faculty of Pharmacy, “Victor Babes” University of Medicine and Pharmacy Timişoara, Eftimie Murgu Square, No. 2, 300041 Timişoara, Romania; cadehelean@umft.ro; 5 Research Center for Pharmaco-Toxicological Evaluations, Faculty of Pharmacy, “Victor Babes” University of Medicine and Pharmacy Timişoara, Eftimie Murgu Square, No. 2, 300041 Timişoara, Romania

**Keywords:** evening primrose oil, cold-pressed oils, natural preservative, lipid peroxidation stability, chorioallantoic membrane

## Abstract

There is a growing need for safer alternatives to synthetic additives commonly used in lipophilic carriers for products such as foods, pharmaceuticals, personal care items, and cosmetics. Natural antioxidants, which prevent lipid peroxidation while providing additional health benefits, offer a promising solution. Evening primrose oil, a rich source of antioxidant compounds with numerous biological benefits, emerges as a potential natural preservative for oil-based products. Our study evaluates a combination of sunflower oil, a widely used cold-pressed oil, with evening primrose oil for potential applications in various fields such as cosmetic, pharmaceutical, or food manufacturing. Various methods were applied to assess oxidative stability by calculating the peroxide value, the *p*-anisidine value, and the total oxidation value, while biological safety was evaluated using the chick embryo’s chorioallantoic membrane and histological analysis. The findings highlight that evening primrose oil, with its balanced effects on epithelial tissues and vascularization, as well as its strong anti-lipid peroxidation properties, is a suitable alternative to synthetic preservatives when used in combination with cold-pressed oils. This proposed oil combination, emphasizing the safety and beneficial properties of evening primrose oil, shows significant potential for applications in the pharmaceutical industry, dermatology, cosmetology, and food manufacturing.

## 1. Introduction

Common evening primrose (*Oenothera biennis* L.) is one of the most abundant species belonging to the *Onagraceae* family [1]. The fruits of the plant are capsules containing numerous seeds from which the oil is extracted [2,3]. Ethnobotanically, there is evidence of the usage of evening primrose as a nutritive and natural remedy for Native American tribes for dermatological diseases, hemorrhoids, menstrual pain, fatigue, and convalescence [3]. Nowadays, there is solid evidence in the scientific literature about the therapeutic activity of *Oenothera biennis* L. cold-pressed seed oil (OBO), showing significant antioxidant, antimicrobial, and anti-inflammatory effects [1,2,4].

Researchers focus on the linkage between the chemical composition of OBO and its biological activity. Regarding the phytochemical constituents, high contents of cis-linoleic acid (70–74%) and cis-*γ*-linolenic acid (8–10%) have been reported [5,6]. The amount of fatty oil extracted by cold-pressing from the seeds is about 24% but varies with age, growth, and cultivation conditions of the plants. Besides the omega-6 essential fatty acids (linoleic acid [LA] and *γ*-linolenic acid [GLA]), evening primrose oil also includes other saturated and unsaturated fatty acids (around 10% oleic acid, 12% palmitic acid, and 2% stearic acid) and 1–2% unsaponifiable matter rich in *β*-sitosterol and campesterol. Other non-triglyceridic constituents of OBO are the linear aliphatic alcohols represented by tetracosanol (236.93 mg/kg oil) and hexacosanol (289.92 mg/kg oil) and the major phenolic compound ferulic acid (25.23 mg/kg oil) [1,7,8]. Additionally, the seeds have been found to contain about 13% protein and 56% carbohydrates such as cellulose and lignin, and as macroelements and microelements, mainly sodium; calcium; potassium; magnesium; iron; and vitamins A, B, C, and E [1,9].

OBO is well known for the treatment of acute and chronic dry skin conditions [10]. The polyunsaturated fatty acids (PUFAs) have shown an enhancement of the epidermal barrier, preventing excessive water loss through the epidermis. OBO also contains polyphenols with anti-inflammatory and antioxidant properties. For the evaluation of the antioxidant potential of OBO, it is essential to determine the peroxide value, the *p*-anisidine value, and the total oxidation number (TOTOX). These parameters provide detailed information on the protective effect of OBO against oxidation processes [11]. Due to its composition rich in antioxidant compounds, besides the various therapeutic benefits, *Oenothera biennis* L. oil can be considered a valuable vegetal product with versatile applications as an ingredient in oil-based products.

Lipophilic carriers are of great importance for several types of products such as foods, pharmaceuticals, personal care products, and cosmetics. The requirements of consumers nowadays imply safe use throughout a long-lasting shelf life. The process of oxidation plays a role in the breakdown of bioactive substances that are beneficial for the human body and generate molecules, some of which can be considered carcinogenic [12]. Furthermore, oxidation degrades and impairs the oil’s sensory characteristics. Lipid oxidation can be successfully prevented by antioxidants. Several approved synthetic preservatives are in use for such purposes. However, the scientific debate regarding safety, in addition to trends in eco-friendly and health-conscious living, is also responsible for the continuous search for safer alternatives to commonly used synthetic additives such as butylated hydroxytoluene (BHT) and replacement with natural antioxidants that prevent lipid peroxidation and, in the meantime, provide additional beneficial effects. Due to their significant antioxidant and antimicrobial effects, different plant extracts and essential oils are evaluated as preservatives for different types of products [13].

Evening primrose oil, as described, represents a rich source of antioxidant compounds, with multiple biological benefits, and could be recommended as an alternative natural preservative for lipophilic products. Our previous investigation regarding the two oils indicated an important content of unsaturated fatty acids, correlated with the protective effect tested oils on 3D tissue models of skin irritation [14]. In SFO, monounsaturated acids (MUFA) were represented by palmitoleic acid (0.158%) and oleic acid (28.249%) as well as the polyunsaturated fatty acids (PUFA), represented by linoleic acid in the highest amount (59.941%) and linolenic acid (0.208%). The same evaluation showed higher levels of PUFA in OBO by 82.247%, mostly linoleic acid (72.093%), arachidonic acid (9.812%), and a significant amount of linolenic acid (0.233%), while that of MUFA was represented by oleic acid, cis form (7.175%), oleic (n10) (0.558%), and 11-eicosenoic (0.210%) [14].

Our study involves the evaluation of a combination between a largely used cold-pressed oil, sunflower oil, with evening primrose oil (SFO-OBO). The high applicability of the proposed combination SFO-OBO especially for human use, either as a food ingredient, or medical or cosmetic administration, requires an evaluation of the lipid peroxidation stability and an assessment regarding the biocompatible properties of the product. For this purpose, multiple methods assessing the oxidative stability were applied, while the biological safety was investigated by using the chick embryo’s chorioallantoic membrane (CAM), a frequently utilized method for various applications, including angiogenic investigations, cancer research, toxicology, bioengineering, and drug development as an in vivo substitute for animal studies [15,16]. The protocol can be successfully employed for detecting biocompatibility with epithelial tissues. The model has several benefits, including cost, time, and accessibility. More precisely, the hen egg test—chorioallantoic membrane assay (HET-CAM), which provides evidence on how a sample can affect the vascular functionality of epithelial tissues, can be used to assess the biocompatibility and irritation potential for different types of samples, including vegetal oils [17]. Moreover, the CAM allows for more in depth investigations into the effects of a sample on the process of angiogenesis, a complex cellular process that consists of the formation of new blood vessels from preexisting ones that are involved in multiple pathologies. The implications in such an important process in health and disease can be explored through microscopic morphology, thus contributing to a wider characterization of the potential in vivo effects, with an impact on the biocompatibility of the samples [18,19].

As a potential alternative use of *Oenothera biennis* L. oil for oil-based products, this study aimed to characterize the combination of OBO with SFO, regarding the oxidative behavior in vitro, in addition to the safety assessment and effects on angiogenesis in ovo.

## 2. Materials and Methods

### 2.1. Oil Samples

OBO was purchased from Mayam, Bucharest, Romania. SFO without added additives was purchased from Solaris, Bucharest, Romania. Both oils are cold-pressed vegetal oils, 100% natural without added additives. The reagents used were purchased from Sigma-Aldrich Chemie GmbH, Munich, Germany, and were of analytical grade. The oils were stored in their original bottles at room temperature (20 ± 2 °C) and kept in their original packaging.

### 2.2. Sample Preparation

Six samples of 50 mL each of SFO without synthetic antioxidants were weighed in different containers. One sample was used as a control, containing only SFO. In the second one, BHT (the maximum legally permitted dose, 200 ppm) was added (SFO–BHT); in the other four, OBO was added in four different concentrations, namely 100, 200, 300, and 500 ppm (SFO–100 OBO, SFO–200 OBO, SFO–300 OBO, and SFO–500 OBO). The obtained oil mixtures were shaken for complete homogenization using a mechanical shaker (Heidolph, Wood Dale, IL, USA).

### 2.3. Determination of Peroxide Value (PV)

PV was evaluated to estimate the antioxidative effect of the addition of OBO to SFO. According to the AOCS Official Method Cd 8-53, the peroxide value of oils represents the quantity of active oxygen (in meq) that can oxidize potassium iodide in one kilogram of fat and was calculated using the iodometric-titration method. PV is expressed in meq O_2_/kg fat [20].

### 2.4. Determination of p-Anisidine Value (p-AV)

To assess the secondary peroxidation products present in the evaluated oil combination, *p*-AV was employed. The official AOCS Cd 18-90 spectrophotometric method [21] was used, and the absorbance of a solution resulting from the reaction of oil in isooctane solution with *p*-anisidine was measured. The method is based on the reaction between the carbonyl group and *p*-anisidine, leading to the formation of an intensely colored Schiff base, which is determined spectroscopically at 350 nm. Thus, 2 g of oil sample was dissolved in 25 mL of isooctane (Sigma-Aldrich Chemie GmbH, Munich, Germany), and the absorbance was read using a UV-VIS spectrophotometer (Specord 205, Analytik Jena Inc., Jena, Germany). The absorbance reading was performed against a control sample consisting of isooctane. Subsequently, 5 mL was taken from the previously prepared solution and was added to 1 mL of 0.25% (*w*/*v*) *p*-anisidine solution in glacial acetic acid (Sigma-Aldrich Chemie GmbH, Munich, Germany). After 10 min, the absorbance was read again at 350 nm. The *p*-AV was calculated using the following Equation (1):

(1) $$p\text{-}\mathrm{AV}=25\;\times \;\frac{1.2\times A_{2}-A_{1}}{W}$$

where A_1_—the absorbance of the fat sample dissolved in isooctane, A_2_—the absorbance of the fat sample dissolved in isooctane + *p*-anisidine solution, and W—weight of the fat sample (g).

### 2.5. Total Oxidation Value (TOTOX)

The total oxidation value indicates the overall oxidation state of oils, determined by the simultaneous assessment of secondary oxidation compounds using AV and primary oxidation products using PV. TOTOX value was calculated using the PV and *p*-AV values according to Equation (2) [22]:

TOTOX value = 2 × PV + AV(2)

The TOTOX value is dimensionless.

### 2.6. In Ovo Evaluation Using the CAM Assay

In order to investigate the effect of the OBO-SFO combination, the in ovo CAM assay was performed. For this purpose, fertilized chicken (*Gallus gallus domesticus*) eggs were incubated in a controlled environment. The protocol included the removal of 4–5 mL of egg white and subsequently performing an upper shell opening [23,24].

### 2.7. Irritation Assessment Using HET-CAM Assay

The chorioallantoic membrane was used here firstly as an alternative protocol to assess the irritation potential of samples as a method of assessing in vivo biocompatibility [17,25]. The protocol used here is an adapted version of the HET-CAM method as recommended by Interagency Coordinating Committee on the Validation of Alternative Methods (ICCVAM) [26].

The fertilized eggs were used on the 9th day of incubation, by applying 300 µL of the control samples, represented by sodium lauryl sulfate (SLS) as a positive control, distilled water as a negative control, or test samples. Evaluation was conducted by means of a stereomicroscope for 300 s, noting the time of occurrence for the following parameters (hemorrhage, H; vascular lysis, L; and coagulation, C) recorded in s. The irritation score (IS) was subsequently determined using the following Equation (3):

(3) $$IS=5\times \left[\frac{301-Sec\;H}{300}\right]+7\times \left[\frac{301-Sec\;L\;}{300}\right]+9\times \left[\frac{301-Sec\;C}{300}\right]$$

To identify the type of irritation the samples might induce, the results expressed as irritation scores were categorized according to Luepke’s scale as 0–0.9—non-irritant, 1–4.9—weak irritant, 5–8.9—moderate irritant, and 9–21—strong irritant [27].

### 2.8. Angiogenesis Evaluation Using the CAM Assay

The samples were also examined in order to investigate the biological reaction of the vascularized CAM following treatment. For this purpose, the samples were applied on the 8th day of incubation, in volumes of 5 µL of solution inside plastic rings previously placed on the CAMs. The effects were examined stereomicroscopically by daily evaluation of the changes observed on the chorioallantoic membranes.

All samples were tested in triplicate. Images for both experiments on the CAM were registered and processed by Axiocam 105 color, AxioVision SE64. Rel. 4.9.1 Software (ZEISS, Göttingen, Germany), ImageJ (ImageJ Version 1.50e, https://imagej.nih.gov/ij/index.html, accessed on 21 November 2024), and GIMP software (GIMP v 2.8, https://www.gimp.org/, accessed on 21 November 2024) [17].

### 2.9. Histological Processing of CAMs

A histopathological assessment of the CAMs was performed at the end of the experiment. For this, the treated areas of the CAMs were excised and immediately fixed in 10% neutral buffered formalin, in order to preserve the tissue structure and prevent degradation. After 24 h, the samples were histoprocessed with a Leica TP1020 Semi-enclosed Benchtop Tissue Processor (Leica Biosystems Nussloch GmbH, Nussloch, Germany), where the tissues were dehydrated using graded series of ethanol, cleared in toluene, and finally soaked in paraffin. Subsequently, using the HistoCore Arcadia H + C Embedding System (Leica Biosystems Nussloch GmbH, Nussloch, Germany), the samples were placed in casting molds, where they were overlaid with melted paraffin for tissue embedding and solidified in a refrigerator for at least 3 h. Subsequently, the hardened blocks containing tissues and the surrounding embedding medium were sectioned using a Leica Biosystems RM2245 Semi-Automated Rotary Microtome (Leica Biosystems Nussloch GmbH, Nussloch, Germany), obtaining 2.5 µm thickness paraffin sections mounted on glass slides. The tissue sections obtained were deparaffinized in toluene and rehydrated with ethanol in decreasing concentrations to distilled water. Afterward, the sections were stained with standard hematoxylin and eosin (HE) stain, dehydrated using ethanol in increasing concentrations, cleared in toluene, and mounted with a resinous medium. Finally, the stained sections were examined by the pathologist in light microscopy using a binocular microscope Leica DM1000 LED (Leica Microsystems AG, Heerbrugg, Switzerland), and the images were taken using a Leica ICC50 W Camera (Leica Microsystems AG, Heerbrugg, Switzerland).

### 2.10. Statistical Analysis

Each determination was performed in triplicate, and the results were presented as mean values ± standard deviation (SD). Results with *p* < 0.05 were considered statistically different. Statistically significant differences were assessed using one-way ANOVA and two-sample *t*-test assuming equal variances. Statistical data processing was performed using Microsoft Excel 2010.

## 3. Results

### 3.1. PV Assessment

In the present study, PV assessment followed the degree of primary oxidation of the studied oil samples during 30 days of storage. Table 1 shows the PV changes in the studied samples. PV was significantly higher in the control sample (SFO) compared to the samples with added BHT and OBO throughout the 30 days of storage.

Thus, as the dose of OBO added to the SFO increased, the PV decreased. The *t*-test performed for the recorded PVs indicated that, compared to day 1, with a normal distribution, the rest of the storage period did not show a regular distribution (*p* < 0.05). According to the *t*-test until day 20, significant differences (*p* < 0.05) were recorded between the PV values of the control sample (SFO) and the samples supplemented with BHT and OEO at 200, 300, and 500 ppm. On days 25 and 30 of storage, significant differences were also recorded between the SFO and the sample supplemented with 100 ppm OBO.

In order to confirm these results, an analysis of variance between samples was also performed using an ANOVA test, and the results obtained led to the same statistical conclusion (*p* < 0.05). Possible differences in the PV of the OBO-supplemented samples (100, 200, 300, and 500 ppm) and the BHT-supplemented sample were also evaluated. Throughout the 30-day storage period, the optimal PV values obtained for SFO–200 BHT were similar to those obtained for SFO–500 OBO, suggesting that a concentration of 500 ppm OBO could substitute the synthetic antioxidant (BHT).

### 3.2. p-AV Assessment

The effect of SFO supplementation with BHT (200 ppm) and OBO (100, 200, 300, and 500 ppm) on *p*-AV during 30 days of storage under ambient conditions is reflected in Table 2.

Following the analysis of the results, *p*-AV was significantly higher in the control sample (SFO) compared to the samples with the addition of BHT and OBO throughout the 30 days of storage. Also, an inverse dose rate effect was observed, by increasing the dose of OBO added to SFO, there was a decrease in *p*-AV.

The *t*-test for *p*-AV showed that there were significant differences between the samples throughout the 30-day storage period (*p* < 0.05). On day 1, there were significant differences between SFO and SFO + OBO (100, 200, and 300 ppm), with no significant differences between SFO + 200 ppm BHT and SFO + 500 ppm OBO. On days 5 and 10, except for SFO + 100 ppm OBO, the other samples showed significant differences compared to SFO. From day 15 to day 30, there were significant differences (*p* < 0.05) between SFO and the rest of the samples supplemented with BHT and OBO. During the whole storage period, there were no significant differences between SFO + 200 ppm BHT and SFO + 500 ppm OBO, suggesting that SFO supplementation with 500 ppm OBO can replace the synthetic antioxidant. Also, in the case of *p*-AV, the results obtained were confirmed by applying the ANOVA test, with the results leading to the same statistical conclusion (*p* < 0.05).

### 3.3. Determination of TOTOX Values

**Figure 1:**
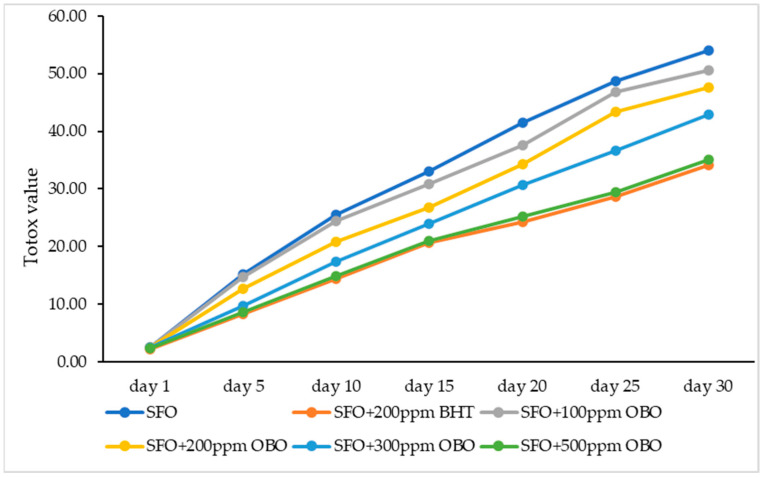
The effect of OBO and BHT on TOTOX of cold-pressed SFO during 30 days of storage period; OBO—*Oenothera biennis* oil, BHT—butylated hydroxytoluene, TOTOX value—total oxidation value, SFO—sunflower oil.

The effect of SFO supplementation with OBO in different concentrations (100, 200, 300, and 500 ppm) on TOTOX values compared to the sample supplemented with 200 ppm BHT is represented in Figure 1. Significantly higher values were recorded for SFO compared to BHT- and OBO-supplemented samples during the 30 days of storage.

Comparing the data presented in Figure 1, the same trend was observed for TOTOX as for PV and *p*-AV. An inverse dose rate effect was maintained. Thus, as the OBO dose increased, the TOTOX values decreased, with SFO + 500 ppm OBO values close to those recorded for SFO + 200 ppm BHT. This confirms that a dose of 500 ppm OBO can substitute the synthetic antioxidant in ensuring the oxidative stability of sunflower oil.

### 3.4. Irritative Potential In Ovo Using HET-CAM Assay

**Figure 2:**
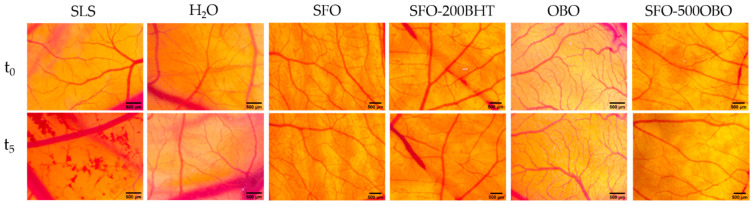
Effects of the tested samples in the HET-CAM assay. Stereomicroscope images show the aspect of CAMs before (t_0_) and after 300 s (t_5_); SLS—sodium lauryl sulfate, OBO—*Oenothera biennis* oil, BHT—butylated hydroxytoluene, SFO—sunflower oil. Scale bars represent 500 µm.

The application of the OBO-containing samples did not induce any change in the evaluated vascular parameters. Compared to the SLS (positive control), which caused a high irritation score of 17.5 ± 0.21 (Table 3), the tested samples, similar to the distilled water (negative control) were all well tolerated, with no irritative effects on the CAM (Figure 2).

### 3.5. Effects on the CAM

**Figure 3:**
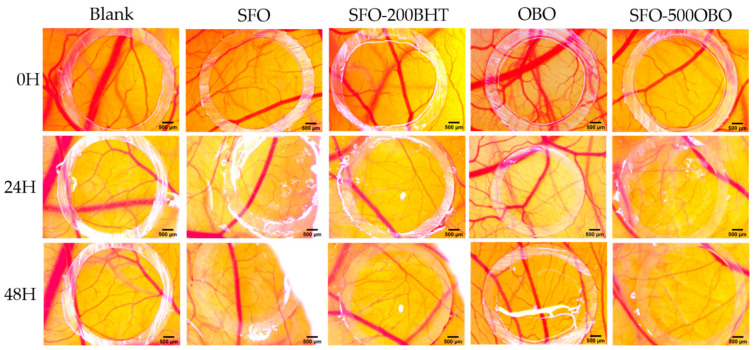
Effect on the CAM of SFO–500 OBO compared to OBO, SFO–200 BHT, SFO, and the blank. Stereomicroscopic images were taken before application, and 24 h and 48 h post-treatment; OBO—*Oenothera biennis* oil, BHT—butylated hydroxytoluene, SFO—sunflower oil. Scale bars represent 500 µm.

The samples were evaluated in an in vivo experimental setting, using the in ovo CAM assay in order to observe the potential biological response on highly vascularized mucosal-like tissues. The evaluation was performed 24 h and 48 h post-treatment. Slight changes upon the normal development of the CAM vascularization were noted on the second day post-inoculation with the samples. More significant modifications regarding the vascular architecture were visible after 48 h. The blank untreated sample displayed on both days post-treatment a normal intense angiogenic process with numerous branches and small capillaries in formation. The cold-pressed SFO did not induce major changes upon the vascular development, with a normal, intense angiogenic process 48 h after treatment. When evaluating the combination between SFO and the two types of preservatives, some changes upon the vascular architecture as compared to the control sample were noted. The combination SFO with the synthetic preservative BHT induced 48 h post-inoculation an overall normal developing CAM vascularization, with some areas inside the application ring having less pronounced branching patterns and some limited hyperemic reactions also observable. The samples containing OBO in concentration of 500 ppm also led to a relatively normal capillary architecture with fine, newly formed vessels observed 48 h post-treatment. The CAMs treated with only OBO showed areas with a more limited number of branches and a narrower aspect of the capillaries, while the combination of SFO and OBO in areas with lower small-vessel density were less significant, showing an overall functional angiogenesis process (Figure 3).

### 3.6. Histological Assessment of CAMs

**Figure 4:**
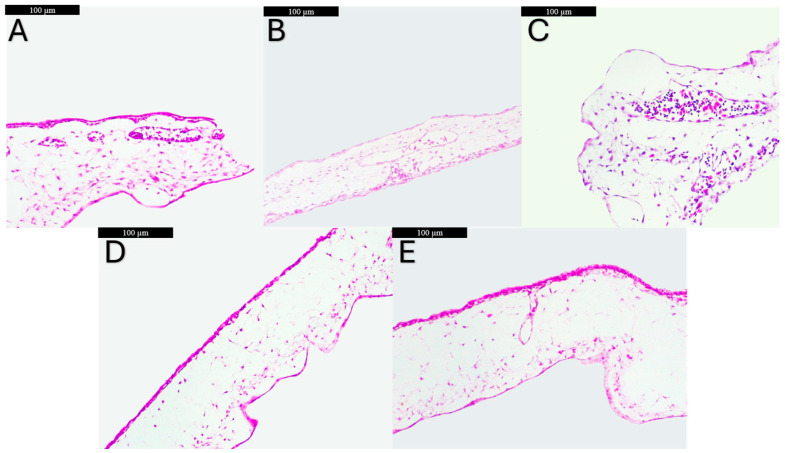
Histological aspects of the CAM treated, HE staining: (**A**) control group with no intervention—normal histological structure; (**B**) SFO CAM group—a slight thinning of the mesodermal layer; (**C**) SFO–BHT CAM group—edema in the mesodermal layer and an increasing influx of intravascular inflammatory cells; (**D**) OBO CAM group—a significant decrease in the number blood vessels in the mesodermal layer; (**E**) SFO–OBO CAM group—a discrete reduction in the number blood vessels in the mesodermal layer; Scale bars represent 100 µm, magnification ×20.

The effect of OBO-containing oils on the CAM was further evaluated from a histological point of view (Figure 4). The CAM specimens from the control group (CAM control) exhibit the normal histological structure presenting the chorionic epithelium, the allantoic epithelium, blood vessels, and fibroblasts in the mesodermal layer (Figure 4A). Compared to the CAM control samples, the application of SFO on the CAM (SFO CAM group) did not significantly affect the tissue architecture, noting only a slight thinning of the thickness of the CAM, by narrowing only the mesodermal layer (Figure 4B). The application of SFO supplemented with BHT (200 ppm) on the CAM (SFO–BHT CAM group) induced a mild inflammatory effect, microscopically observing the thickening of the CAM—through edema at the mesodermal level and an increasing intravascular influx of inflammatory cells (Figure 4C). The treatment with OBO on the CAM (OBO CAM group) determined architectural change at the mesodermal level, with a significant decrease in the number of blood vessels standing out (Figure 4D), while the application of SFO supplemented with OBO (500 ppm) on CAM (SFO–OBO CAM group) determined only a discrete reduction in the number of blood vessels, but without the narrowing, thinning of the mesodermal layer (Figure 4E).

## 4. Discussion

Our study explored the potential benefits of using an alternative preservative for oil-based products, investigating the evening primrose oil, known for its active compounds such as tocopherols and phenols with antioxidant properties [28], in combination with sunflower oil. The oxidative stability of SFO combined with OBO was evaluated compared to those of pure SFO and SFO with BHT. The primary and secondary oxidative compounds produced during storage were assessed by calculating PV, AV, and TOTOX. PV is an important indicator in quality control and safety assessment of oil-based products that provide information on the level of hydroperoxide as the primary oxidation products of fat and oils formed due to the oxidation process in the initial stage of fat degradation [22]. Thus, hydroperoxide generation must be avoided since oxidized compounds are typically harmful [29].

Throughout the 30-day storage period, similar PV values for SFO–200 BHT were obtained for SFO–500 OBO, suggesting that a concentration of 500 ppm OBO can supplement the synthetic antioxidant. A similar trend was reported by Kamkar et al. in their study on the antioxidant effect of *Mentha pulegium* L. essential oil on SFO [30]. Also, Cocan et al. studied the inhibitory effect on the oxidation of SFO in the case of its supplementation with chili and sweet pepper seed oil, showing an effect similar to BHT [22]. Raza et al. studied the inhibitory effect of *Centella asiatica* L. essential oil on the oxidation of SFO during 7 days of storage in ambient storage conditions. They concluded that supplementation of SFO at a concentration of 200 ppm had the same inhibitory effect as another chemical antioxidant, butylated hydroxyanisole (BHA), throughout the storage period [31].

Moreover, *p*-AV values provide information on the degradation state of lipids by the amount of aldehydes formed during the autoxidation process as by-products [32]. The results obtained in the present study are promising for the establishment of a new alternative antioxidative combination for sunflower oil. Other similar studies in the literature use different types of vegetal antioxidant mixing ingredients. Thus, Wang et al. recorded a similar trend when supplementing SFO with essential oil from *Punica granatum* cv. Heyinshiliu peel [33]. Also, Cocan et al. obtained similar results in the study on the inhibitory effect of chili and sweet pepper seed oil on the oxidation of SFO, indicating possible alternatives to BHT [22]. Wang et al. studied the inhibitory effect of *Coriandrum sativum* L. essential oil on oxidation occurring in sunflower oil during 24 days of storage in ambient storage conditions, showing for a concentration of 1200 ppm the same inhibitory effect as tert-butyl hydroquinone (TBHQ) throughout the storage period [34].

In order to obtain the most accurate results in terms of oxidative stability, it is essential to examine the evolution of primary (PV) and secondary (*p*-AV) oxidation by calculating the total oxidation of the samples [35]. The TOTOX assay confirms once again that a dose of 500 ppm OBO can substitute the synthetic antioxidant in ensuring the oxidative stability of sunflower oil. A similar trend was also reported by others using essential oils or chili oils as supplementation for SFO, indicating decreased TOTOX values for different concentrations [22,33]. Evening primrose crude oil was previously reported as a tocopherol and phenolic compound-rich oil with good antioxidative properties [28]. The supplementation of SFO with evening primrose was investigated in another study that employed ethyl acetate extract, which expressed a better antioxidative activity than BHT (0.01%), extending the oil’s shelf-life [36]. Our group also previously analyzed the oxidative stability of SFO when supplemented with OBO by using the thiobarbituric acid-reactive substances assay (TBA), indicating good oxidative stability [14]. No other studies were found using evening primrose oil for the supplementation of sunflower oil.

For the in vivo biological and tolerability assessment of the tested oil combinations, CAM assays were employed as useful and convenient alternatives to animal testing [16]. Firstly, the irritability evaluation was performed using the HET-CAM method, which allows for the estimation of the degree of negative impact upon vascularized epithelial tissues. All evaluated samples were totally unharmful on the CAM, thus pointing towards the multiple cosmetic, pharmaceutical, or food uses of the proposed new combination of sunflower oil with evening primrose oil up to the concentration of 500 ppm. A good tolerability was also exhibited by the traditionally used chemical antioxidant BHT in a concentration of 200 ppm in combination with SFO, in accordance with other studies. Other formulations, mainly emulsions [37] or nanoemulsions [38] containing BHT in variable concentrations were tested by others, indicating that the tested products were non-irritant up to 0.5% of BHT, a higher concentration than the 200 ppm considered for the present study. Still, no available data were found regarding the irritability of a simple combination between cold-pressed oils and BHT. OBO is well known for its skin anti-inflammatory and regenerative benefits [1], and its tolerability was confirmed by skin patch studies [39]. Our group investigated in vitro the mixture of SFO and OBO on 3D tissue models of skin irritation and phototoxicity, showing good biocompatibility and no toxic effects [14]. We managed to use for the present study a simple, cost-efficient assay, alternatively to animal testing, and the results indicate a good tolerability, being of significant value as preliminary data for further tests, ensuring also the safety of the future participants, patients, or consumers.

Secondly, we used the same biological material to evaluate the effect of the test combination on the angiogenesis process of the CAM. The cold-pressed SFO did not induce major changes upon vascular development, with a normal, intense angiogenic process. This effect may be caused by the high content of LA and low GLA in SFO [40,41]. LA was reported to promote angiogenesis [42,43]. Meanwhile, for the combination between SFO and the two types of preservatives, some changes upon the vascular architecture as compared to the control sample were noted. The combination of SFO with the synthetic preservative BHT induced a normally developing CAM vascularization, but with a more limited branching pattern and a limited peripheric hyperemic reaction. In terms of angiogenesis and cancer promotion, the effect of BHT is controversial [44]; it was recently reported to limit angiogenesis by targeting COX-2, HOXA-10, and MMP-9 [45]. The samples containing OBO alone led to a relative normal vascularization and small areas with narrower capillaries, while for the combination SFO–OBO, the areas with lower small-vessel density were limited, with no alteration of the angiogenic process. The effect may be partly due to the presence of significant concentrations of GLA considered as a key active component of OBO [5,46], which was reported to possess anti-inflammatory and anti-angiogenic effects in vivo after oral administration [47]. Based on these facts, it is possible that the combination of SFO—having a high LA:GLA ratio—with OBO—a major source of GLA—expresses an overall more neutral effect during the angiogenesis process, in contrast with the more pronounced reduction in the branching pattern, in addition to the hyperemic effect after incubation with the SFO–BHT combination.

A more in-depth evaluation was performed by morphological examination of HE staining of the vascularized CAMs exposed to the oil combinations. The changes arising in all the histological structures of the CAM were observed after the topical application of the tested samples. SFO treatment on the CAM caused only a slight narrowing of the mesodermal layer of the CAM and no negative impact on the angiogenic process, as observed stereomicroscopically, thus allowing the normal intense angiogenic process characteristic for the embryonic age (7–9 embryonic developmental days) [15], whereas the treatment with SFO supplemented with BHT on the CAM, as visualized macroscopically, induced signs of inflammation—edema in the mesodermal layer and an increased cellular influx in the blood vessels, which suggests a potential inflammatory effect of BHT at 200 ppm. The application of OBO alone on the CAM was well tolerated with no irritation signs, showing only a moderately reduced number of blood vessels in the mesodermal layer, in agreement with other studies evaluating OBO and GLA [1,47], while the application of SFO supplemented with OBO (500 ppm) led to a decrease in the number of blood vessels to a lesser extent as compared to the SFO–BHT combination, without causing thinning of the mesoderm layer, thus suggesting the combination SFO–OBO as an optimal solution with good tolerability on epithelial tissues.

The morphological changes induced by the application of OBO were predominantly observed at the level of the mesodermal layer, the embryological precursor of the dermis, thus explaining the improvement in skin parameters such as restoration of the epidermal skin barrier, normalization of the excessive trans-epidermal water loss, and smoothness improvement, after topical application [48]. The valuable pharmacological properties of OBO provide directions for its incorporation into various cosmeceutical skincare products, like moisturizers, ointments that prevent skin dryness and irritation, and antiaging and antiphotoaging lotions. The protective effect of OBO on skin, linked to the high content in omega-3 fatty acids, being especially a significant source of GLA, is also consistent with other data [1,4,14,49].

Our study addresses the challenges of developing safer, stable formulations that retain the desired properties of natural oils without the need for additional synthetic stabilizers. In this view, using evening primrose oil as an alternative preservative for oil-based products, in combination with sunflower oil, can be envisaged as a valuable option for a wide range of products with good oxidative stability, lack of irritative effects, and good tolerability. Still, to overcome skepticism about the performance and safety of natural oils, such as evening primrose oil, compared to synthetic preservatives, more extensive evaluations and cost-efficiency analyses are required.

## 5. Conclusions

Our findings suggest that OBO, through its balanced effect on epithelial tissues and vascularization and an important anti-lipid peroxidation effect, represents a more suitable ingredient compared to the synthetic BHT, in combination with SFO or other cold-pressed oils. Combining evening primrose oil with sunflower oil offers a promising preservative alternative, providing good oxidative stability, minimal irritation, and excellent tolerability for a variety of products in diverse applications. Future research should focus on in-depth performance testing, thus paving the way for their wider implementation in industries such as pharmaceuticals, cosmetics, and food production.

## Figures and Tables

**Table 1:** The effect of OBO and BHT on PV of cold-pressed SFO during 30 days of storage.

Sample	PV (meq O_2_/kg Oil)						
	Storage Period (Days)						
	Day 1	Day 5	Day 10	Day 15	Day 20	Day 25	Day 30
SFO	1.125 ± 0.033 ^a,A^	4.228 ± 0.127 ^a,B^	7.705 ± 0.231 ^a,C^	9.846 ± 0.359 ^a,D^	12.442 ± 0.456 ^a,E^	15.224 ± 0.554 ^a,F^	16.458 ± 0.569 ^a,G^
SFO–BHT	1.050 ± 0.025 ^a,A^	3.345 ± 0.098 ^d,B^	5.568 ± 0.165 ^d,C^	8.058 ± 0.288 ^c,D^	9.228 ± 0.374 ^d,E^	10.861 ± 0.376 ^d,F^	12.246 ± 0.428 ^e,G^
SFO–100 OBO	1.122 ± 0.032 ^a,A^	4.118 ± 0.125 ^a,B^	7.221 ± 0.225 ^a,C^	9.507 ± 0.327 ^a,D^	12.105 ± 0.426 ^a,E^	14.804 ± 0.488 ^b,F^	15.551 ± 0.527 ^b,G^
SFO–200 OBO	1.118 ± 0.030 ^a,A^	3.872 ± 0.120 ^b,B^	6.824 ± 0.214 ^b,C^	9.016 ± 0.312 ^b,D^	11.755 ± 0.408 ^b,E^	14.169 ± 0.472 ^b,F^	15.024 ± 0.508 ^c,G^
SFO–300 OBO	1.113 ± 0.028 ^a,A^	3.567 ± 0.110 ^c,B^	6.229 ± 0.208 ^c,C^	8.721 ± 0.296 ^b,D^	11.207 ± 0.391 ^c,E^	13.275 ± 0.416 ^c,F^	14.473 ± 0.476 ^d,G^
SFO–500 OBO	1.111 ± 0.027 ^a,A^	3.389 ± 0.104 ^d,B^	5.705 ± 0.189 ^d,C^	8.119 ± 0.292 ^c,D^	9.633 ± 0.385 ^d,E^	11.132 ± 0.380 ^d,F^	12.583 ± 0.451 ^e,G^

OBO—*Oenothera biennis* oil, BHT—butylated hydroxytoluene, PV—peroxide value, SFO—sunflower oil. All results are expressed as mean values ± SD. The *t*-test was used to show significant differences between the values recorded for the samples; letters with different superscripts (a–e) in the same column represent significant differences between different samples (*p* < 0.05); and letters with different superscripts (A–G) in the same row represent significant differences recorded for the same sample analyzed on different days, maintained at room temperature (*p* < 0.05).

**Table 2:** The effect of OBO and BHT on *p*-AV of cold-pressed SFO during 30 days of storage.

Sample	*p*-AV						
	Storage Period (Days)						
	Day 1	Day 5	Day 10	Day 15	Day 20	Day 25	Day 30
SFO	0.225 ± 0.006 ^a,A^	6.742 ± 0.201 ^a,B^	10.074 ± 0.301 ^a,C^	13.423 ± 0.421 ^a,D^	16.627 ± 0.557 ^a,E^	18.297 ± 0.594 ^a,F^	21.183 ± 0.676 ^a,G^
SFO–BHT	0.159 ± 0.003 ^b,A^	1.669 ± 0.048 ^d,B^	3.341 ± 0.100 ^d,C^	4.547 ± 0.122 ^e,D^	5.824 ± 0.199 ^e,E^	6.993 ± 0.257 ^e,F^	9.725 ± 0.331 ^e,G^
SFO–100 OBO	0.221 ± 0.006 ^a,A^	6.529 ± 0.151 ^a,B^	9.943 ± 0.297 ^a,C^	11.826 ± 0.396 ^b,D^	13.309 ± 0.413 ^b,E^	17.246 ± 0.561 ^b,F^	19.552 ± 0.611 ^b,G^
SFO–200 OBO	0.212 ± 0.005 ^a,A^	4.884 ± 0.114 ^b,B^	7.182 ± 0.208 ^b,C^	8.734 ± 0.287 ^c,D^	10.776 ± 0.359 ^c,E^	15.055 ± 0.488 ^c,F^	17.506 ± 0.562 ^c,G^
SFO–300 OBO	0.204 ± 0.004 ^a,A^	2.546 ± 0.069 ^c,B^	4.930 ± 0.167 ^c,C^	6.525 ± 0.203 ^d,D^	8.228 ± 0.284 ^d,E^	10.119 ± 0.307 ^d,F^	13.892 ± 0.417 ^d,G^
SFO–500 OBO	0.168 ± 0.003 ^b,A^	1.840 ± 0.054 ^d,B^	3.441 ± 0.101 ^d,C^	4.669 ± 0.157 ^e,D^	5.931 ± 0.188 ^e,E^	7.197 ± 0.263 ^e,F^	9.932 ± 0.305 ^e,G^

OBO—Oenothera biennis oil, BHT—butylated hydroxytoluene, *p*-AV—*p*-anisidine value, SFO—sunflower oil. All results are expressed as mean values ± SD. The *t*-test was used to show significant differences between the values recorded for the samples; letters with different superscripts (a–e) in the same column represent significant differences between different samples (*p* < 0.05); and letters with different superscripts (A–G) in the same row represent significant differences recorded for the same sample analyzed on different days, maintained at room temperature (*p* < 0.05).

**Table 3:** Irritation scores and type effects induced using the HET-CAM assay.

Samples	Irritation Score	Type of Effect
SLS 0.5%	17.50 ± 0.21	strong irritant
H_2_O dist.	0 ± 0.0	non-irritant
SFO	0 ± 0.0	non-irritant
SFO–BHT	0 ± 0.0	non-irritant
OBO	0 ± 0.0	non-irritant
SFO–500 OBO	0 ± 0.0	non-irritant

SLS—sodium lauryl sulfate, OBO—Oenothera biennis oil, BHT—butylated hydroxytoluene, SFO—sunflower oil. All results are expressed as mean values ± SD.

## Data Availability

The original contributions presented in this study are included in the article. Further inquiries can be directed to the corresponding author.

## References

[1] Timoszuk M., Bielawska K., Skrzydlewska E. (2018). Evening Primrose (*Oenothera biennis*) Biological Activity Dependent on Chemical Composition. Antioxidants.

[2] Heinrich M., Barnes J., Gibbons S., Williamson E. (2012). Fundamentals of Pharmacognosy and Phytotherapy.

[3] Steckel L.E., Sosnoskie L.M., Steckel S.J. (2019). Common Evening-Primrose (*Oenothera biennis* L.). Weed Technol..

[4] Fecker R., Buda V., Alexa E., Avram S., Pavel I.Z., Muntean D., Cocan I., Watz C., Minda D., Dehelean C.A. (2020). Phytochemical and Biological Screening of *Oenothera biennis* L. Hydroalcoholic Extract. Biomolecules.

[5] Farag M., Reda A., Nabil M., Elimam D., Zayed A. (2023). Evening Primrose Oil: A Comprehensive Review of Its Bioactives, Extraction, Analysis, Oil Quality, Therapeutic Merits, and Safety. Food Funct..

[6] Hadidi M., Ibarz A., Pouramin S. (2021). Optimization of Extraction and Deamidation of Edible Protein from Evening Primrose (*Oenothera biennis* L.) Oil Processing by-Products and Its Effect on Structural and Techno-Functional Properties. Food Chem..

[7] Ghatas Y., Mohamed Y. (2020). Influence of Some Phosphorus Sources and Biofertilizers (Em and Phosphorein) on Vegetative Growth, Fixed Oil Productivity and Chemical Constituents of *Oenothera biennis* L. Plant. Sci. J. Flowers Ornam. Plants.

[8] Montserrat-De La Paz S., Fernández-Arche M.A., Ángel-Martín M., García-Giménez M.D. (2014). Phytochemical Characterization of Potential Nutraceutical Ingredients from Evening Primrose Oil (*Oenothera biennis* L.). Phytochem. Lett..

[9] Wang Z., Wu Z., Zuo G., Lim S.S., Yan H. (2021). Defatted Seeds of *Oenothera biennis* as a Potential Functional Food Ingredient for Diabetes. Foods.

[10] Edwards S., Rocha I., Williamson E., Heinrich M. (2015). Phytopharmacy: An Evidence-Based Guide to Herbal Medical Products.

[11] Frankel E.N. (2005). Lipid Oxidation.

[12] Nowak K., Jabłońska E., Ratajczak-Wrona W. (2021). Controversy around Parabens: Alternative Strategies for Preservative Use in Cosmetics and Personal Care Products. Environ. Res..

[13] Tinello F., Lante A. (2020). Accelerated Storage Conditions Effect on Ginger- and Turmeric-Enriched Soybean Oils with Comparing a Synthetic Antioxidant BHT. LWT.

[14] Fecker R., Magyari-Pavel I.Z., Cocan I., Alexa E., Popescu I.M., Lombrea A., Bora L., Dehelean C.A., Buda V., Folescu R. (2022). Oxidative Stability and Protective Effect of the Mixture between *Helianthus annuus* L. and *Oenothera biennis* L. Oils on 3D Tissue Models of Skin Irritation and Phototoxicity. Plants.

[15] Nowak-Sliwinska P., Segura T., Iruela-Arispe M.L. (2014). The Chicken Chorioallantoic Membrane Model in Biology, Medicine and Bioengineering. Angiogenesis.

[16] Tamanoi F. (2019). Recent Excitements in the Study of the CAM Assay. The Enzymes.

[17] Avram Ș., Bora L., Vlaia L.L., Muț A.M., Olteanu G.-E., Olariu I., Magyari-Pavel I.Z., Minda D., Diaconeasa Z., Sfirloaga P. (2023). Cutaneous Polymeric-Micelles-Based Hydrogel Containing Origanum Vulgare L. Essential Oil: In Vitro Release and Permeation, Angiogenesis, and Safety Profile In Ovo. Pharmaceuticals.

[18] Avram S., Ghiulai R., Pavel I., Mioc M., Babuta R., Voicu M., Coricovac D., Danciu C., Dehelean C., Soica C. (2017). Phytocompounds Targeting Cancer Angiogenesis Using the Chorioallantoic Membrane Assay. Natural Products and Cancer Drug Discovery.

[19] Minda D., Ghiulai R., Banciu C.D., Pavel I.Z., Danciu C., Racoviceanu R., Soica C., Budu O.D., Muntean D., Diaconeasa Z. (2022). Phytochemical Profile, Antioxidant and Wound Healing Potential of Three Artemisia Species: In Vitro and In Ovo Evaluation. Appl. Sci..

[20] (2009). AOCS Official Method Cd 8b-90, Peroxide Value Acetic Acid-Isooctane Method. Official Methods and Recommended Practices of the AOCS.

[21] (2017). AOCS Official Method Cd 18-90, p-Anisidine Value. Official Methods and Recommended Practices of the AOCS.

[22] Cocan I., Negrea M., Cozma A., Alexa E., Poiana M., Raba D., Danciu C., Popescu I., Cadariu A.I., Obistioiu D. (2021). Chili and Sweet Pepper Seed Oil Used as a Natural Antioxidant to Improve the Thermo-Oxidative Stability of Sunflower Oil. Agronomy.

[23] Ghiulai R., Avram S., Stoian D., Pavel I.Z., Coricovac D., Oprean C., Vlase L., Farcas C., Mioc M., Minda D. (2020). Lemon Balm Extracts Prevent Breast Cancer Progression In Vitro and In Ovo on Chorioallantoic Membrane Assay. Evid.-Based Complement. Altern. Med..

[24] Ribatti D. (2010). The Chick Embryo Chorioallantoic Membrane as an in Vivo Assay to Study Antiangiogenesis. Pharmaceuticals.

[25] Scheel J., Kleber M., Kreutz J., Lehringer E., Mehling A., Reisinger K., Steiling W. (2011). Eye Irritation Potential: Usefulness of the HET-CAM under the Globally Harmonized System of Classification and Labeling of Chemicals (GHS). Regul. Toxicol. Pharmacol..

[26] National Institute of Environmental Health Sciences (2010). ICCVAM Test Method Evaluation Report: Current Validation Status of In Vitro Test Methods Proposed for Identifying Eye Injury Hazard Potential of Chemicals and Products.

[27] Luepke N.P. (1985). Hen’s Egg Chorioallantoic Membrane Test for Irritation Potential. Food Chem. Toxicol..

[28] Pan F., Li Y., Luo X., Wang X., Wang C., Wen B., Guan X., Xu Y., Liu B. (2020). Effect of the Chemical Refining Process on Composition and Oxidative Stability of Evening Primrose Oil. J. Food Process. Preserv..

[29] Gotoh N., Wada S. (2006). The Importance of Peroxide Value in Assessing Food Quality and Food Safety. JAOCS J. Am. Oil Chem. Soc..

[30] Kamkar A., Javan A.J., Asadi F., Kamalinejad M. (2010). The Antioxidative Effect of Iranian Mentha Pulegium Extracts and Essential Oil in Sunflower Oil. Food Chem. Toxicol..

[31] Ali Raza S., Rehman A., Adnan A., Qureshi F. (2009). Comparison of Antioxidant Activity of Essential Oil of Centella Asiatica and Butylated Hydroxyanisole in Sunflower Oil at Ambient Conditions. Biharean Biol..

[32] Skiera C., Steliopoulos P., Kuballa T., Holzgrabe U., Diehl B. (2012). 1H NMR Approach as an Alternative to the Classical P-Anisidine Value Method. Eur. Food Res. Technol..

[33] Wang D., Meng Y., Zhao X., Fan W., Yi T., Wang X. (2019). Sunflower Oil Flavored by Essential Oil from Punica Granatum Cv. Heyinshiliu Peels Improved Its Oxidative Stability and Sensory Properties. LWT.

[34] Wang D., Fan W., Guan Y., Huang H., Yi T., Ji J. (2018). Oxidative Stability of Sunflower Oil Flavored by Essential Oil from *Coriandrum sativum* L. during Accelerated Storage. LWT—Food Sci. Technol..

[35] Domínguez R., Pateiro M., Gagaoua M., Barba F.J., Zhang W., Lorenzo J.M. (2019). A Comprehensive Review on Lipid Oxidation in Meat and Meat Products. Antioxidants.

[36] Niklová I., Schmidt Š., Habalová K., Sekretár S. (2001). Effect of Evening Primrose Extracts on Oxidative Stability of Sunflower and Rapeseed Oils. Eur. J. Lipid Sci. Technol..

[37] Ermawati D., Cahyani P.Z., Shahnaz I., Juniarty A., Mahardhika C.L., Chasanah U. (2023). Formulation of Serum Using a Combination of Tamanu Oil and Tea Tree Oil as Anti-Acne. J. Kefarmasian Indones..

[38] Bernardi D.S., Pereira T.A., Maciel N.R., Bortoloto J., Viera G.S., Oliveira G.C., Rocha-Filho P.A. (2011). Formation and Stability of Oil-in-Water Nanoemulsions Containing Rice Bran Oil: In Vitro and in Vivo Assessments. J. Nanobiotechnol..

[39] Kaniuk Ł., Podborska A., Stachewicz U. (2022). Enhanced Mechanical Performance and Wettability of PHBV Fiber Blends with Evening Primrose Oil for Skin Patches Improving Hydration and Comfort. J. Mater. Chem. B.

[40] Akkaya M.R. (2018). Prediction of Fatty Acid Composition of Sunflower Seeds by Near-Infrared Reflectance Spectroscopy. J. Food Sci. Technol..

[41] Harun M. (2019). Fatty Acid Composition of Sunflower in 31 Inbreed and 28 Hybrid. Biomed. J. Sci. Tech. Res..

[42] Samson F.P., Patrick A.T., Fabunmi T.E., Yahaya M.F., Madu J., He W., Sripathi S.R., Tyndall J., Raji H., Jee D. (2020). Oleic Acid, Cholesterol, and Linoleic Acid as Angiogenesis Initiators. ACS Omega.

[43] Nishioka N., Matsuoka T., Yashiro M., Hirakawa K., Olden K., Roberts J.D. (2011). Linoleic Acid Enhances Angiogenesis through Suppression of Angiostatin Induced by Plasminogen Activator Inhibitor 1. Br. J. Cancer.

[44] Bauer A.K., Dwyer-Nield L.D., Hankin J.A., Murphy R.C., Malkinson A.M. (2001). The Lung Tumor Promoter, Butylated Hydroxytoluene (BHT), Causes Chronic Inflammation in Promotion-Sensitive BALB/CByJ Mice but Not in Promotion-Resistant CXB4 Mice. Toxicology.

[45] Sun Z., Gao R., Chen X., Liu X., Ding Y., Geng Y., Mu X., Liu T., Li F., Wang Y. (2021). Exposure to Butylated Hydroxytoluene Compromises Endometrial Decidualization during Early Pregnancy. Environ. Sci. Pollut. Res..

[46] Evening Primrose Oil. https://pubchem.ncbi.nlm.nih.gov/compound/Evening-Primrose-Oil.

[47] Andreoli Miyake J., Nascimento Gomes R., Colquhoun A. (2020). Gamma-Linolenic Acid Alters Migration, Proliferation and Apoptosis in Human and Rat Glioblastoma Cells. Prostaglandins Other Lipid Mediat..

[48] Muggli R. (2005). Systemic Evening Primrose Oil Improves the Biophysical Skin Parameters of Healthy Adults. Int. J. Cosmet. Sci..

[49] Munir R., Semmar N., Farman M., Ahmad N.S. (2017). An Updated Review on Pharmacological Activities and Phytochemical Constituents of Evening Primrose (*Genus oenothera*). Asian Pac. J. Trop. Biomed..

